# Trouble

**Published:** 1882-01

**Authors:** J. B. Chandler


### "TROUBLE."

J. B. CHANDLER.

Providence is blamed for so ordering that human beings shall all have trouble; and men quietly submit to what they deem inevitable. If they have a physical ailment, they immediately set about having it cured, but trouble is nursed and petted; men indulge in the luxury of woe and brood over their cares until everything, trifling or real, is magnified into unendurable sorrow and "enterprises of great pith and moment with this regard, their currents turn away and lose the name of action."

Certainly much that we trouble ourselves about is imaginary, or at least our dwelling upon it does no good. Many persons put on their thoughts and actions a gloom which they oftentimes assume in compliance with custom, but which eventually colors their inmost thoughts and kills out all that would make life enjoyable. This naturally follows, because the proper digestion and assimilation of food demands a cheerful mind.

It follows, then, that the less trouble we entertain, or rather, as trouble seems inevitable to man "as sparks fly upward," the easier we bear trouble the sooner it will pass off, and the more strength we shall have to contend with the next unwelcome visitor.

Let us consider some of the troubles which we take most to heart and mourn longest over. As good Christians, we believe that this life is but a state of probation, from whence the good go to meet eternal reward, freed from all pain and trouble. Yet we stand, day by day, night after night, sometimes for months, watching by the bedside of some loved relative who has probably passed through the period of life in which we enjoy, we watch him suffering agony until death comes to his relief—then, do we rejoice that his troubles are over? No; we immediately decorate ourselves in the panoply of woe, close up our houses, darken our rooms, participate in no pleasures, and "refuse to be comforted." A child dies, escaping from the cares and dangers of life, going to One who said, "Suffer little children to come unto me, for of such is the kingdom of heaven." Few griefs are greater than parents feel at such a time. Yet what earthly pleasure that they have ever experienced could they offer their child in the place of the joys the Saviour promises?

In these two instances of what are considered real troubles, has not education, habit, fashion, something to do with our grief? What good can our crape and solemn faces do the loved ones gone, or in what way enable us to overcome the the loss of their association?

Suppose we had been taught to rejoice at the good going to their reward as we make merry at a wedding—the latter may be but the beginning of bitter trouble to those we rejoice over, yet we dance and give thanks, whilst custom enjoins the opposite for the loved one relieved of earth's pains and cares. Petty annoyances are allowed to work us up into frenzy sometimes, when a little calm contemplation might result in putting all quietly to rest without any interference with life's duties and pleasures.

What will the world say? What will Mrs. Grundy say about this or that deviation from set rules, is another source of trouble which would soon cease if people would only attend to their own business in their own way, doing right as best they can. Why get a new cloak or a new bonnet, when the old ones are comfortable and decent, and you cannot really afford it, just because somebody else has a new style! Why worry and fret because your best actions are misconstrued by others, who perhaps would not act as well under similar circumstances? Yet we all do worry, and do buy new clothes when old ones would answer, just because fashion and a false sensibility demand it. In other words, most of our troubles are imaginary, and would soon depart before sensible reflection. There are morbid states produced by sickness, dyspepsia and other diseases, which give rise to imaginary troubles that, from the weak state of the body, are less easily overcome. Yet the remedy is not wanting. Dyspepsia is not incurable, and with the disappearance of the disease, the mental trouble will become amenable to reason.

The French proverb, "*A quelque chose malheur est bon*," is true. There is scarcely a misfortune in which may not eventually be found a blessing. We may fail to see it at the time, but it comes in due season. It seems, then, that we have the power to make our lives happy within ourselves, in spite of outside troubles. A determined stand of a few against foolish customs will soon bring about their abandonment and make us see with clearer vision, capable of discerning a joy where we now find a sorrow. I would not advocate a harsh disregard for the prejudices and opinions of our neighbors, but the time has come when people are beginning to throw off the fetters of old habits and inquire into the why and wherefore of things. Notwithstanding the advance of late in æsthetic taste, still we are becoming more practical, and perhaps the day is not far distant when men will not mourn when they should rejoice, nor sing pæans of joy when the case is one of doubtful result.

